# Evaluating Effects of Divided Hemispheric Processing on Word Recognition in Foveal and Extrafoveal Displays: The Evidence from Arabic

**DOI:** 10.1371/journal.pone.0018131

**Published:** 2011-04-29

**Authors:** Abubaker A. A. Almabruk, Kevin B. Paterson, Victoria McGowan, Timothy R. Jordan

**Affiliations:** College of Medicine, Biological Sciences, and Psychology, University of Leicester, Leicester, United Kingdom; Royal Holloway, University of London, United Kingdom

## Abstract

**Background:**

Previous studies have claimed that a precise split at the vertical midline of each fovea causes all words to the left and right of fixation to project to the opposite, contralateral hemisphere, and this division in hemispheric processing has considerable consequences for foveal word recognition. However, research in this area is dominated by the use of stimuli from Latinate languages, which may induce specific effects on performance. Consequently, we report two experiments using stimuli from a fundamentally different, non-Latinate language (Arabic) that offers an alternative way of revealing effects of split-foveal processing, if they exist.

**Methods and Findings:**

Words (and pseudowords) were presented to the left or right of fixation, either close to fixation and entirely within foveal vision, or further from fixation and entirely within extrafoveal vision. Fixation location and stimulus presentations were carefully controlled using an eye-tracker linked to a fixation-contingent display. To assess word recognition, Experiment 1 used the Reicher-Wheeler task and Experiment 2 used the lexical decision task.

**Results:**

Performance in both experiments indicated a functional division in hemispheric processing for words in extrafoveal locations (in recognition accuracy in Experiment 1 and in reaction times and error rates in Experiment 2) but no such division for words in foveal locations.

**Conclusions:**

These findings from a non-Latinate language provide new evidence that although a functional division in hemispheric processing exists for word recognition outside the fovea, this division does not extend up to the point of fixation. Some implications for word recognition and reading are discussed.

## Introduction

Although unilateral projections to each contralateral hemisphere are well-established for each visual hemifield [1]–[3], the projection of information around the point of fixation has recently become a matter of considerable debate in word recognition research [4], [5]. A longstanding view is that an area of foveal vision (up to 3 degrees wide) exists around the point of fixation within which information projects (bilaterally) to both hemispheres, and a functional division in hemispheric projection occurs only outside this area (for relevant evidence and discussions, see [4]–[17]). In recent years, however, some researchers have promoted the contrasting view that a clear and functional division in hemispheric projection occurs right up to the point of fixation because each human fovea is divided so precisely at its vertical midline that even adjacent letters in a word that fall either side of fixation project unilaterally to different contralateral hemispheres (see [4], [5], [11], [12], [18]–[27]). Thus, according to this view, all information to the left of fixation will project unilaterally to the right hemisphere (RH), and all information to the right of fixation will project unilaterally to the left hemisphere (LH). Moreover, since it is well-established that the LH generally has superior perceptual capabilities for words than the RH (see [28] for a review), this putative division in hemispheric processing at the point of fixation is claimed to have important effects on word recognition [18]–[27].

Advocates of this split-fovea view have attempted to reveal evidence of split-foveal processing by observing the effect on word recognition of presenting stimuli at various eccentricities around the point of fixation (for a review and critical discussion, see [4], [5]). For example, a typical approach has been to present words at offsets to the left or right of a fixation point so that they straddle this point at various locations (and in some studies these words are also shown entirely to the right or left of this point in nearby locations; [18], [24]–[27]). The findings have shown a word recognition advantage when most of the letters in a word, or words in their entirety, were shown to the right of the fixation point. Thus, according to these studies, word recognition was determined by the hemisphere to which the letters presented to the left and right of fixation were projected, and a processing advantage was produced when most or all of these letters were presented to the right of the fixation point because all letters to the right of fixation projected to the LH.

The logic of this approach is that if word recognition is affected by a division in unilateral, contralateral hemispheric projections right up to the point of fixation, this division will be revealed by asymmetries in performance for words displayed to the left and right of fixation even when stimuli are displayed close to fixation and entirely within foveal vision. However, two requirements are fundamental to the success of this approach. First, it is crucial that participants in studies of split-foveal processing fixate the designated fixation point with sufficient accuracy to ensure that information presented either side of this point is actually presented at the correct location in the appropriate hemifield. Second, it is crucial that stimuli are of an appropriate size to always be displayed entirely within foveal vision away from areas of known unilateral contralateral projection. Without these controls, apparent effects of split-foveal processing can be confounded by the presence of visual information in the wrong location in each hemifield (and even in the wrong hemifield), or by the presence of visual information in extrafoveal locations where the existence of highly-influential contralateral projections is already well established [9]. Unfortunately, the vast majority of studies conducted in support of split-foveal processing fail to adhere to either of these requirements (for a description of the substantial problems that exist in previous research in this area, see [4], [5]). Moreover, studies that have used appropriate fixation and stimulus control show no evidence of a functional division in hemispheric projections for words displayed in foveal vision even though providing clear evidence of a functional division in hemispheric processing for words displayed to the right and left of fixation in extrafoveal locations. For example, several experiments have used fixation-contingent displays to present words precisely in matched locations each side of fixation, either entirely within foveal or extrafoveal vision [29], [30]. This approach provides the most straightforward test of the split-fovea view, by assessing whether left-right differences in the recognition of words displayed at extrafoveal locations (where hemispheric asymmetries in word recognition are well-established and not contentious) extend all the way to the point of fixation and so are also observed for words entirely displayed in foveal vision at locations to the left and right of fixation. These experiments produced a strong recognition advantage for words presented to the right of fixation in extrafoveal vision but not for the same words presented to the right of fixation in foveal vision. The findings are therefore consistent with a functional division in hemispheric projections for words encountered outside foveal vision but indicate no functional division for words within foveal vision. Other studies using appropriate fixation and stimulus control and a variety of paradigms and procedures have also found no evidence of split-fovea processing (for reviews, see [4], [5] and Footnote 1 in File S1).

Such findings are clearly problematic for accounts of word recognition based on split-foveal processing. However, in line with other research in this area, previous research using appropriate fixation and stimulus control to investigate split-foveal processing has been conducted using Latinate languages which, as we will describe, may militate against the discovery of evidence to support split-foveal processing. As a result, it remains to be seen whether, under precisely-controlled experimental conditions, alphabetic languages with properties fundamentally different from Latinate languages can provide evidence of a functional division in hemispheric processing in foveal word recognition.

Arabic has the second most widely-used alphabet in human societies, after the Latin alphabet. Moreover, like Latinate languages, Arabic produces perceptual superiority for words displayed to the right of fixation at extrafoveal locations, indicating classic LH dominance for processing words in this language [31]. However, Arabic is notably absent from split-fovea research.

Importantly for our purposes, the characteristics of Arabic differ fundamentally from those of Latinate languages [32]. In particular, text in Arabic is read from right to left, but text in Latinate languages is read from left to right and the beginning (leftmost) letters of words are unusually important for word recognition [33]–[38]; (see also [39]–[43]). Consequently, when words in Latinate languages are displayed to the left and right of fixation in experiments, beginning letters are closer to fixation when displayed to the right and the difference this causes in the visibility of beginning letter information may help produce a LH advantage (for further discussion, see [41]). More importantly, this left-right difference in beginning letter visibility between the two visual hemifields would be greater for stimuli further from fixation, and this may explain why LH advantages observed previously for words in extrafoveal locations have not also been observed in foveal locations [29], [30]. However, Arabic is read from right to left, and the importance of beginning letters in Arabic for determining word identity is much less, due to the nonconcatenative derivational morphology of Arabic [32], [44]–[52]. Indeed, letters that are of special importance for word recognition are distributed throughout words in Arabic rather than concentrated towards the beginning (as in Latinate languages). As a consequence, Arabic should allow a particularly transparent investigation of a functional division in hemispheric processing of words in foveal and extrafoveal displays.

In addition, letters in Latinate languages are typically highly distinct, especially for words displayed close to fixation. Thus, when words in these languages are presented in lateralised displays in experiments, indications of a functional division in hemispheric processing may not be apparent for foveal stimuli because letter discriminability in these locations is so high [4], [5]. In contrast, Arabic is formed in a cursive script which decreases the distinctiveness of individual letters in words and introduces additional crowding [53], [54] that may further decrease letter resolution (for discussions of difficulty in letter perception in Arabic, see [47], [49]). Consequently, Arabic stimuli may provide challenging displays that are well-suited to revealing a functional division in hemispheric processing for foveal word recognition, should this exist.

In line with these considerations, two experiments were conducted to assess the recognition of Arabic words displayed in the left and right visual hemifields at locations either close to fixation and entirely in foveal vision or further from fixation and entirely in extrafoveal vision. Both experiments used appropriate fixation and stimulus control [4], [5]. Previous research that has investigated hemispheric influences on recognition of Arabic words [31] (and other languages read from right to left [55]–[59]) indicates LH superiority for words displayed at extrafoveal locations. Consequently, if a functional division in hemispheric processing exists right up to fixation, performance should be superior for words displayed to the right of fixation in extrafoveal *and* foveal locations. If, however, a functional division in hemispheric processing occurs only outside the fovea, performance should be superior for words displayed to the right of fixation in extrafoveal locations but not foveal locations, even under the experimental conditions afforded by Arabic stimuli.

## Methods

### Ethics Statement

This research was conducted with the ethical approval of the School of Psychology Ethics Committee at the University of Leicester, and in accordance with the ethical guidelines of the British Psychological Society. All participants gave informed consent in writing.

### Experiment 1: The Reicher-Wheeler task

Experiment 1 used the Reicher-Wheeler task [60], [61] to assess performance. In this procedure, brief displays of words are followed by a forced choice between two alternatives that differ by one (critical) letter. For example, if “word” was displayed as the target, “word” and “work” may then be displayed as alternatives and participants would be required to indicate which alternative had been displayed as the target. Across the experiment, all letter positions are tested and all alternatives are presented as targets. The primary benefit of the Reicher-Wheeler task is that it reveals processes of word recognition while suppressing influences of artefactual bias based on partial word information because the correct response containing the critical letter (in this case “word”) cannot be deduced from other parts of the stimulus (w-o-r) [41]–[43], [62]–[64]. Thus, and in line with the concerns raised earlier, while participants may be more able to guess a word's identity when presented to one side of fixation simply because parts of words can be seen more readily, the Reicher-Wheeler task will suppress left-right imbalances in the informativeness of partial word information and, when combined with the benefits of using Arabic stimuli, will provide a particularly powerful technique for assessing word recognition in the left and right hemifields.

#### Participants

Twelve native Arabic speakers took part. All had normal or corrected to normal visual acuity, determined using a Bailey-Lovie eye chart, and were right-handed, determined by a revised Annett Handedness questionnaire [65]. Eye dominance was determined individually using the hole in the card test [66], [67].

#### Stimuli

120 five-letter Arabic words from the Aralex database [68] and 120 matched five-letter Arabic pseudowords were used. Following the requirements of the Reicher–Wheeler task, words were selected to form matched pairs in which the members of each pair differed by just one, critical letter (e.g., the Arabic words 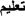 and 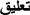, which differ at the final, leftmost, letter position) and these differences occurred equally often at each of the five letter positions across all stimuli. Pseudoword stimuli were constructed for each pair by re-arranging the 4 non-critical letters in each word to form pronounceable pseudowords. An additional 12 five-letter words and 12 five-letter pseudowords were constructed to provide 24 practice stimuli at the beginning of each session. The same practice stimuli were displayed at the beginning of each session.

**Figure 1:**
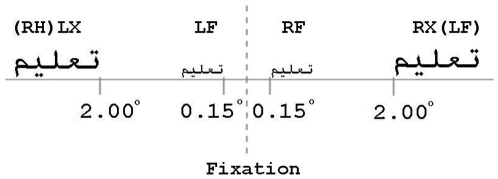
Screen locations of foveal and extrafoveal stimuli in Experiments 1 and 2. Footnote: The size of foveal and extrafoveal stimuli was matched for visibility. The terms RH and LH refer to the hemisphere contralateral to the hemifield in which stimuli were presented.

Stimuli were presented in standard cursive Arabic script as black text on a white background at foveal and extrafoveal locations to the left and right of a central fixation point (see Figure 1). The physical size of the stimuli presented at foveal and extrafoveal locations was adjusted to avoid confounding effects of visibility on overall levels of performance [29], [30], [69] and to ensure that stimuli were shown entirely in either foveal or extrafoveal locations. Accordingly, foveal stimuli subtended approximately 1° horizontally, and the inner edges of these stimuli were 0.15° from fixation. Extrafoveal stimuli subtended approximately 2° horizontally, and the inner edges of these stimuli were 2° from fixation. Preliminary testing established that these sizes and eccentricities produced similar levels of overall performance for foveal and extrafoveal displays.

#### Apparatus

Stimuli were presented on a high-definition display and a Cambridge Research Systems VSG 2/5 card controlled stimulus presentations. Responses were collected via a Cambridge Research Systems CT3 response box. The experiment was conducted in a sound-attenuated and darkened room. Stimulus viewing was monocular via each participant's dominant eye to eliminate confounding effects of binocular fixation disparity [70] and each non-dominant eye was occluded using a light-proof eye-patch (Cambridge Research Systems). The fixation location of each dominant eye was monitored using a Skalar IRIS eye-tracking system (Cambridge Research Systems) clamped to each participant's head, and this in turn was clamped in a head brace and chin rest throughout the experiment to prevent movement. The output of the eye-tracker was recorded through the ADC input of the VSG 2/5 card and this arrangement allowed accurate and consistent measurement of fixation location to within 5 minutes of arc (for further details, see [29], [30], [71]).

#### Design

Participants took part in 3 sessions, one on each of 3 different days. Within each session, words and pseudowords were selected pseudo-randomly and assigned pseudo-randomly to the four stimulus locations. Across all sessions, each participant was shown 960 experimental presentations (320 in each session) so that each experimental word and pseudoword was shown once in each stimulus location.

#### Procedure

At the start of each session, participants were given instructions describing the forced-choice task and emphasising the importance of accuracy when responding. The eye-tracker was then calibrated. At the start of each trial, a single but clearly visible pixel (the fixation point) was presented at the centre of the screen. Participants were required to fixate this point and stimulus display was prevented until accurate fixation occurred continuously for 300 ms. Once this criterion was satisfied, a stimulus was presented for 33 ms at one of the four stimulus locations. If fixation deviated from the fixation point before stimulus presentation, stimulus presentation was immediately prevented and continued to be prevented until accurate fixation occurred again for at least 300 ms (for further details of this procedure, see [71], [72]).

Immediately following each display, the target stimulus and its matched pair-mate were displayed one above the other, in random order, and participants indicated which had been shown by pressing the appropriate key on the response box. The fixation point then reappeared for the next target display. The alternatives were presented in a size intermediate between the two sizes used for target presentations, at the bottom of the screen well away from the locations at which targets were presented, and were displayed until a response was made. Hand of response was counterbalanced across participants, so that half the participants responded with their right hand and half responded with their left hand.

### Experiment 2: The Lexical Decision Task

Advocates of the split-fovea account have criticised previous research that used the Reicher-Wheeler task and which found no evidence of an effect of split-foveal processing on word recognition by suggesting that it is an off-line task that does not provide reaction times measures of performance and so may not be sensitive to small effects associated with split-foveal processing of words [19], [73]. However, the task is clearly sensitive to left-right divisions in hemispheric processes of word perception when these divisions occur (i.e., for extrafoveal locations) but provides no evidence at all for split-foveal processing, as both the present experiment and previous research have demonstrated [29], [30]. The Reicher-Wheeler task has the particular advantage of being specifically designed to suppress influences of bias that might otherwise create spurious indications of the influence of hemispheric asymmetries on word recognition (for further discussion, see [29], [30], [41], [42], [74]. However, other techniques can be used if they are selected carefully and conducted appropriately. For example, some researchers have used overt naming to assess word recognition performance in studies of hemispheric asymmetry using Latinate languages [25], [75]. Unfortunately, overt naming is problematic because speech production in the vast majority of individuals is lateralised to the LH and so naming can produce a spurious advantage for information projected to the LH because this information is projected to the hemisphere responsible for producing a response rather than because this hemisphere is superior for recognizing that information. However, not all tasks suffer from this problem and one paradigm that has been used widely in word recognition research is the lexical decision task which requires stimuli to be identified either as words or pseudowords as quickly and as accurately as possible. This task does not provide the same control offered by the Reicher-Wheeler task and may not be as sensitive to processes of perception [76]. Nevertheless, it has been shown to be sufficiently sensitive to reveal hemispheric asymmetries in word recognition when hand of response is appropriately counterbalanced to avoid hemispheric confounds in responding [77].

Therefore, to extend the findings of Experiment 1, Experiment 2 investigated the influence of a division in hemispheric processing on perception of Arabic words and pseudowords in foveal and extrafoveal locations using a lexical decision task with appropriate stimulus and fixation control and counterbalanced hand of response. As in Experiment 1, the predictions were straightforward: If a functional division in hemispheric processing exists right up to fixation, performance should be superior for words displayed to the right of fixation in extrafoveal *and* foveal locations. If, however, a functional division in hemispheric processing occurs only outside the fovea, performance should be superior for words displayed to the right of fixation in extrafoveal locations but not foveal locations.

#### Participants

Twelve native Arabic speakers took part. All had normal or corrected to normal vision, determined using a Bailey-Lovie eye chart, and were right-handed, determined by a revised Annett Handedness questionnaire [65], and had not taken part in Experiment 1. Eye dominance was determined individually using the hole in the card test [66], [67].

#### Procedure

At the start of each session, participants were given instructions describing the lexical decision task and emphasising the importance of speed and accuracy when responding. On each trial, a target word or pseudoword was shown for 150 ms at one of the four stimulus locations. Participants were required to decide whether the stimulus was a word or pseudoword and to press the appropriate key on the response box, one marked “word” and the other marked “pseudoword”. Hand of response was counterbalanced across participants, so that half of the participants responded with their right hand and half responded with their left hand, and all used their index finger to make responses. All other aspects of this experiment, including design, stimuli, and apparatus, were identical to those of Experiment 1.

## Results and Discussion

### Experiment 1: The Reicher-Wheeler task

#### Results

**Figure 2:**
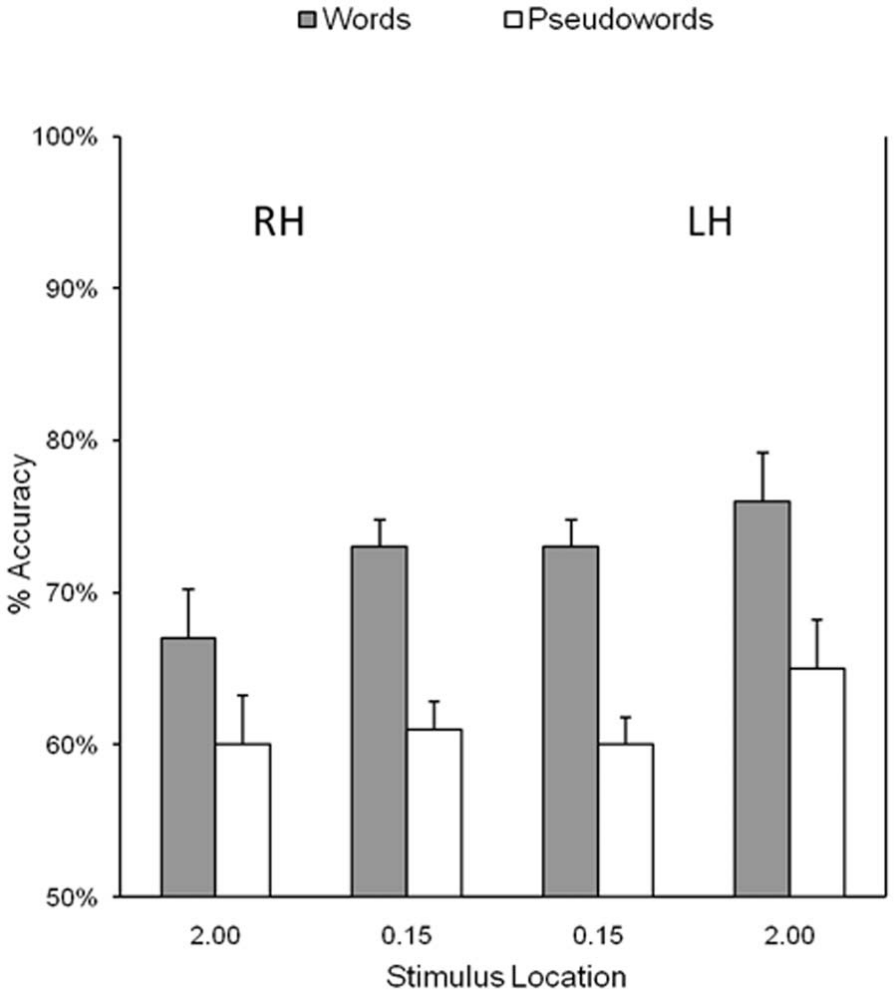
Results for Experiment 1. Footnote: Mean identification accuracy for Arabic words and pseudowords displayed at foveal and extrafoveal locations in Experiment 1. Bars correspond to 95% confidence intervals [78]. The terms RH and LH refer to the hemisphere contralateral to the hemifield in which stimuli were presented.

Mean identification accuracy for words and pseudowords displayed at foveal and extrafoveal locations is shown in Figure 2 (bars indicate the 95% confidence interval for each mean based on the within subjects mean square error [78]). Preliminary analyses showed no significant effect of response hand or session and these variables were not included in subsequent analyses. As the physical size of stimuli presented at foveal and extrafoveal locations in the experiment was adjusted to avoid confounding effects of visibility on performance, comparisons of performance between foveal and extrafoveal locations were not of theoretical interest. Consequently, a 2 (lexicality: words vs. pseudowords)×2 (hemisphere: left vs. right) repeated measures ANOVA was performed on responses to stimuli separately for foveal and extrafoveal locations.

#### Accuracy for foveal displays

There was a main effect of lexicality (words = 73%, pseudowords = 60%), *F*(1,11) = 19.78, *p* = .001, η_p_^2^ = .64, but no effect of hemisphere (*F*<1.5) or interaction between these factors (*F*<1.3). Indeed, stimuli displayed in foveal vision showed no indication of a division in hemispheric processing (words, RH = 73%, LH = 73%; pseudowords, RH = 61% LH = 60%).

#### Accuracy for extrafoveal displays

Main effects were found for lexicality (words = 72%, pseudowords = 62%), *F*(1,11) = 22.50, *p*<.001, η_p_^2^ = .67, and hemisphere (RH = 63%, LH = 71%), *F*(1,11) = 16.33, *p*<.002, η_p_^2^ = .60, with no interaction between these factors (*F*<1.5), indicating an LH advantage for words and pseudowords (words, 67% vs. 76%; pseudowords, 60% vs. 65%) (see Footnote 2 in File S1).

#### Discussion

Experiment 1 showed that Arabic words in extrafoveal locations were recognised more accurately when displayed to the right of fixation. In line with previous research, this finding provides further evidence for superior LH recognition of Arabic words [31]. However, no evidence of this hemispheric asymmetry was observed for foveal word recognition. In fact, word recognition was equally accurate either side of fixation in foveal vision, providing no support for the split-foveal view that a functional division in hemispheric processing exists up to the point of fixation.

Although not the focus of the present research, pseudowords also showed evidence of an LH advantage for extrafoveal displays but again no indication of a functional division in hemispheric processing for foveal displays. Previous research using Latinate languages [43], [79], [80] has also indicated an LH advantage for pseudowords in extrafoveal locations and, like that research, our findings also showed a slightly smaller advantage for pseudowords than for words (5% vs. 9%). Previous research has accounted for these findings in terms of a general LH processing advantage for legal letter-strings which becomes even greater for stimuli with lexical representations, and our findings are consistent with this view.

### Experiment 2: The Lexical Decision Task

#### Results

**Figure 3:**
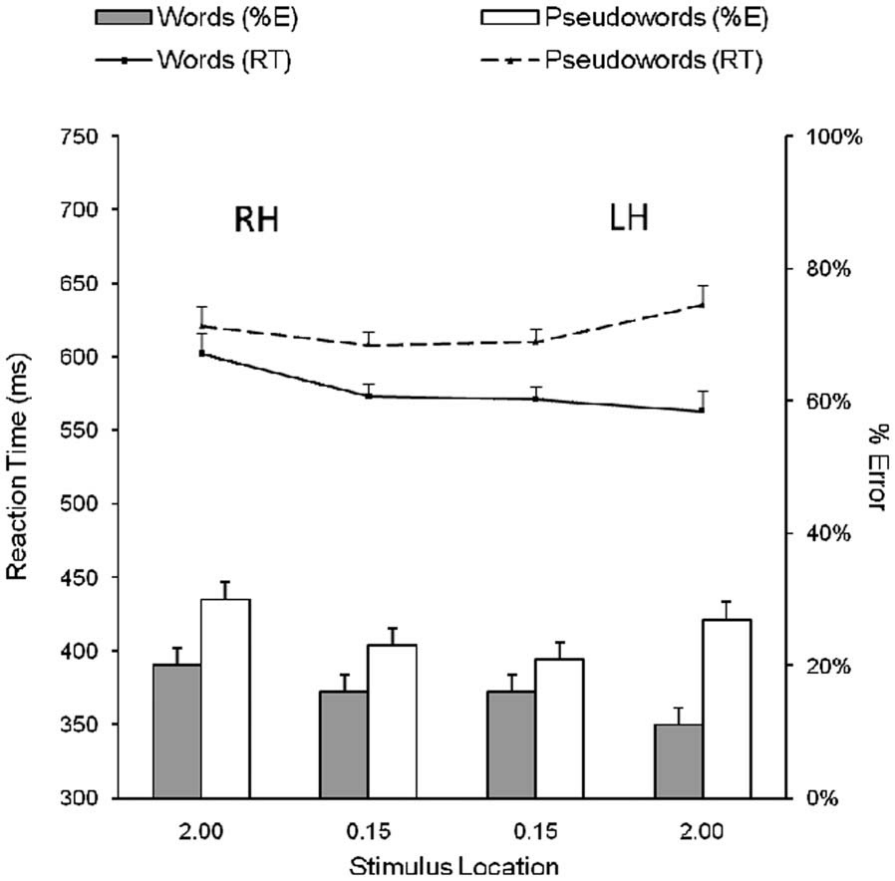
Results for Experiment 2. Footnote: Mean reaction times for correct responses and error rates for Arabic words and pseudowords displayed at foveal and extrafoveal locations in Experiment 2. Bars correspond to 95% confidence intervals [78]. The terms RH and LH refer to the hemisphere contralateral to the hemifield in which stimuli were presented.

Mean reaction times for correct responses and error rates for words and pseudowords displayed at foveal and extrafoveal locations are shown in Figure 3 (bars indicate the 95% confidence interval for each mean based on the within subjects mean square error [78]). Preliminary analyses showed no significant effect of response hand or session and these variables were not included in subsequent analyses. As in Experiment 1, because the physical size of stimuli presented at foveal and extrafoveal locations was adjusted to avoid confounding effects of visibility on performance, comparisons of performance between foveal and extrafoveal locations were not of theoretical interest. Consequently, a 2 (lexicality: words vs. pseudowords) ×2 (hemisphere: left vs. right) repeated measures ANOVA was performed on reaction times and error rates separately for foveal and extrafoveal locations.

#### Reaction times for foveal displays

There was a main effect of lexicality (words = 572 ms, pseudowords = 609 ms), *F*(1,11) = 10.13, *p*<.01, η_p_^2^ = .48, but no main effect of hemisphere or an interaction (*F*s<1). Consequently, there was no indication of a division in hemispheric processing for stimuli displayed in foveal vision (words, RH = 573 ms, LH = 570 ms; pseudowords, RH = 608 ms, LH = 610 ms).

#### Error rates for foveal displays

There was no main effect of lexicality, *F*(1,11) = 3.60, *p* = .09, η_p_^2^ = .25, or hemisphere, *F*(1,11) = 1.81, *p* = .21, η_p_^2^ = .14, or an interaction between these factors (*F*<1.5), and so no indication of a division in hemispheric processing for stimuli displayed in foveal vision (words, RH = 16%, LH = 16%; pseudowords, RH = 23%, LH = 21%).

#### Reaction times for extrafoveal displays

There were main effects of lexicality (words = 582 ms, pseudowords = 628 ms), *F*(1,11) = 40.49, *p*<.001, η_p_^2^ = .79, and hemisphere (LH = 599 ms, RH = 611 ms), *F*(1,11) = 4.72, *p* = .05, η_p_^2^ = .30, and an interaction between these factors, *F*(1,11) = 15.52, *p*<.01, η_p_^2^ = .59. A strong LH advantage was observed for words (602 ms vs. 563 ms, *p*<.01) but not for pseudowords (621 ms vs. 635 ms, *p*>.05).

#### Error rates for extrafoveal displays

There was a main effect of lexicality (words = 16%, pseudowords = 29%), *F*(1,11) = 22.88, *p*<.001, η_p_^2^ = .68, but no main effect of hemisphere (*F*<1.5). However, there was an interaction between these factors, *F*(1,11) = 17.57, *p*<.002, η_p_^2^ = .30. A strong LH advantage was observed for words (20% vs. 11%, *p*<.01) and a similar pattern for pseudowords, although this was not significant (30% vs. 27%, *p*>.05) (see Footnote 3 in File S1).

#### Discussion

The findings from Experiment 2 showed that words in extrafoveal locations were recognised more quickly and more accurately when displayed to the right of fixation, and so provide further evidence of an LH advantage for processing Arabic words. But again there was no evidence of a similar division in hemispheric processing for words displayed in foveal locations. The findings for words in both experiments, therefore, provide strong evidence of hemispheric division in word recognition for extrafoveal, but not foveal, displays. Moreover, it is clear that these findings are not task specific since they were obtained using the two very different paradigms provided by the Reicher-Wheeler task and the lexical decision task.

#### General Discussion

The experiments reported in this article investigated the view that a precise split in human foveae at the vertical midline causes all information presented to the left or right of fixation to project unilaterally to the contralateral hemisphere [18]–[27]. As a consequence, influences of hemispheric asymmetry on word recognition well-established for words presented either side of fixation in extrafoveal locations should occur right up to the point of fixation, and so should also be observed for words presented either side of fixation in foveal vision.

The validity of the split-fovea view was assessed using Arabic stimuli in two experiments, one using the Reicher-Wheeler task (Experiment 1) and one the lexical decision task (Experiment 2). Both experiments used fixation and stimulus controls required for an accurate assessment of split-foveal processing, and appropriate counterbalancing of hand of response to avoid spurious indications of hemispheric asymmetries in word recognition. Performance in both tasks showed an LH advantage for words in extrafoveal locations (in recognition accuracy in Experiment 1 and in reaction times and error rates in Experiment 2) but no hemispheric advantage for foveal locations in either experiment. (Pseudowords also showed an LH advantage for extrafoveal displays in Experiment 1, and no hemispheric advantage for foveal displays in either experiment). These findings support the well-established view that the LH is specialised for word recognition in alphabetic languages and provides further evidence that an LH advantage also occurs for languages, such as Arabic, that are read from right to left [31], [55]–[59]. However, this asymmetry in word recognition was observed only for extrafoveal displays and no indication of a functional division in hemispheric processing at the point of fixation was observed [4], [29], [30].

This finding is consistent with the longstanding view that an area exists about the foveal midline within which ipsilateral and contralateral projections are intermingled and so information in this area projects bilaterally to both hemispheres (for relevant evidence and discussions, see [6]–[17], [81]). The precise extent of this area of overlap remains to be determined (and may be supported by rapid interhemispheric communication [82]) but bilateral projections appear to provide benefits for visual processing generally by supporting binocular vision around the point of fixation and enabling continuity in vision across the foveal midline [17], [81]. Moreover, a particular advantage of bilateral processing for foveal word recognition is that it enables the letters of words to be processed equally effectively regardless of where these letters fall within the fovea. For example, in Latinate languages, the beginnings of words are unusually important for word recognition [33]–[37] and so it would be particularly advantageous for this information to be projected to the LH. However, when a word in a Latinate language is fixated, this beginning letter information will usually fall to the left of fixation. Consequently, if a precise division in hemispheric processing did exist at the point of fixation, this split would cause beginning letter information to project unilaterally to the RH. This would produce a paradoxical consequence of split-foveal processing since information that is of special importance for word recognition in Latinate languages would project to the hemisphere that has least efficient word processing capabilities. In textual reading of Latinate languages, partial pre-processing of words to the right of fixation may provide some pre-activation of LH processing. However, the impediment of divided hemispheric projections when words are fixated (either individually or during reading) would be avoided by bilateral projections because all information from a word encountered in this bilateral region would be projected to the LH as well as to the RH. Indeed, the problem may also be avoided even if contralateral and ipsilateral hemispheric projections in human foveae are split anatomically but the processing of words in foveal vision was functionally bilateral because transmission of information between the two hemispheres was sufficiently rapid to obviate a functional role for an anatomical divide when subsequent processes of word recognition eventually became active [82]. In this case, hemispheric division in early visual processing may still produce functionally bilateral projections for processes of word recognition within foveal vision.

Bilateral projections may also benefit foveal word recognition in Arabic, and other languages that are read from right to left. Split-foveal processing in these languages would not produce the paradox of projecting beginning letter information to the RH because, when a word is fixated, beginning letters would now fall to the right of fixation, and so project to the LH (if the split-fovea view were correct). However, because of the different morphological construction of words in these languages compared to Latinate languages, the important consideration for word recognition in Arabic (and other Semitic languages) is the efficient identification of a word's morphological root [31], [32], [46], [48], [50]–[52], [83], [84].

In particular, the vast majority of words in Semitic languages such as Arabic are created from triliteral roots that comprise a sequence of three consonants that express the general meaning of a word and combine with other letters (which form the word pattern) to create different inflections of meaning. For example, the Arabic root comprising the consonants 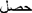 combines with other letters to form words such as 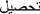. In Latinate languages, morphological composition is achieved by affixation, whereby adding a morpheme as the prefix or suffix of a word creates the desired inflectional meaning. However, Semitic languages have a nonconcatenative morphology in which the root and word pattern do not combine via affixation, and the letters of these two components intermingle to form a word. For example, in the word 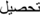, the root consonants appear as the second, third, and final letters and are combined with other letters that form the word pattern. Consequently, the root is not identifiable as a contiguous sequence of letters, and must be identified from a sequence of consonants spread throughout the word [45], and this may have important consequences for word recognition [50]–[52]. Indeed, it is of particular relevance to the present research that when a word in Arabic is fixated, consonants that form the root are unlikely to project to retinal locations on the same side of fixation. Consequently, if hemispheric processing is divided at the point of fixation, these consonants will often project to different hemispheres and recognition of the root may be delayed until the letters are recombined via interhemispheric transfer. By comparison, bilateral foveal processing of words in these languages has the capacity to achieve greater efficiency in word recognition by ensuring that all the letter information needed to identify the root is made available rapidly to each hemisphere regardless of which side of the foveal midline this information occurs. In a similar vein, split-fovea processing also presents problems for processing exterior letters of words in Latinate languages, which have a privileged role as a unified feature in word recognition and provide valuable information about the physical length of a word and word identity [40], [85], [86]. Consequently, a split in foveal processing at the point of fixation would cause the exterior letters of fixated words to project to different hemispheres and valuable information provided by conjoint exterior letter features may be lost (see Footnote 4 in File S1).

The implications of a division in hemispheric processing for foveal Arabic word recognition may also impact on even the basic processing of Arabic words. Several studies indicate that the RH is particularly poor at identifying Arabic letters [31], [47], [87], and this may be exacerbated by the poorer discriminability of individual letters in words and additional crowding [54] introduced by the cursive nature of Arabic script. The explanation given for this deficiency is that the RH may have a specific difficulty with Arabic letters, due to letters sharing the same basic form and to the extensive use of dots to mark distinctions between letters in Arabic script. For example, the Arabic letters representing /t/ and /n/ (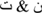) become the graphemes that represent /th/ and /b/ (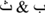), respectively, simply by adding or changing the number or location of small dots within the word. Split-foveal accounts require that asymmetries in hemispheric processing of words extend right up to the point of fixation, and so this particular RH deficiency in Arabic processing should affect recognition of Arabic words to the left of fixation in foveal and extrafoveal vision (and, indeed, recognition of those parts of fixated Arabic words that fall to the left of fixation). However, from the experiments reported here, recognition of Arabic words is not affected by such a catastrophic division in foveal processing. In particular, both experiments showed no evidence of this asymmetry in performance for Arabic words displayed in foveal locations but clear asymmetries when these words were presented in extrafoveal locations. The indications are, therefore, that a division in projection to impoverished RH letter processing occurs for extrafoveal, but not foveal, word recognition.

The present research shows clear evidence of a division in hemispheric processing for extrafoveal Arabic word recognition but it remains to be determined what influences these asymmetries have on the processing of words in locations outside the fovea to the right and left of fixation during reading. Little is known about the benefits of parafoveal processing for reading in Arabic but it is well established in Hebrew and Latinate languages that information about the next word or words in a sentence is acquired during reading and that this information is used to pre-process the identity of these words and to programme eye movements (for an overview of this and other research on eye movements during reading, see [88]–[92]). However, the precise influence of hemispheric projections on parafoveal processing of words in textual reading is unknown (but see [93]–[95]). One likely possibility is that the nature of the parafoveal processing that takes place will reflect the language processing abilities of the hemisphere to which this information projects. Consequently, for most readers in Latinate languages, parafoveal processing of words to the right of fixation (i.e., in the direction of reading), will take advantage of the superior word recognition capabilities of the LH to which these words would project unilaterally. In contrast, qualitatively different effects may be observed in reading Arabic, where parafoveal processing of words to the left of fixation (i.e., in the direction of reading) would involve unilateral projections to the inferior language processing capabilities of the RH. The effects of these differences in hemispheric projection on reading efficiency have yet to be revealed.

In sum, the findings reported in this article provide new evidence that a functional division in hemispheric processing for word recognition exists outside the fovea but not at the point of fixation. Importantly, these findings were obtained using Arabic stimuli with properties fundamentally different from the Latinate languages that are typically the focus of split-fovea research, and fixation and stimulus control that provided appropriate levels of precision for investigating the issue of split-fovea processing. Moreover, the findings were obtained across two very different paradigms (the Reicher-Wheeler task and the lexical decision task), indicating that a functional division in hemispheric processing for Arabic words in extrafoveal but not foveal locations is not task specific. Consequently, although a functional division in hemispheric processing at the point of fixation is fundamental to the split-foveal processing view, the mounting evidence obtained with appropriate experimental precision from Latinate and now Arabic languages provides no indication that such a division exists.

## Supporting Information

File S1 (DOC)
