# Supplementary material for: Evaluating Effects of Divided Hemispheric Processing on Word Recognition in Foveal and Extrafoveal Displays: The Evidence from Arabic
Source: PLoS One. 2011 Apr 29;6(4):e18131. doi: 10.1371/journal.pone.0018131 (PMC3084692; doi:10.1371/journal.pone.0018131)
Supplement: File S1 — (DOC) [file pone.0018131.s001.doc]

Footnotes

1. Recent assessments of fixation accuracy in studies of split-fovea processing [29,96,97] confirm long-held concerns [9,98-102] over the frequency and extent of fixation inaccuracies that occur when participants are simply instructed where to fixate or are given a secondary fixation task at the required point of fixation. Indeed, substantial fixation inaccuracy has been reported on approximately 50-80% of trials in experiments using these weak fixation controls. However, it should be noted that, despite the weight of this evidence, one study reporting support for split-fovea processing [73] and which also found fixation inaccuracies on 65% (Experiment 2) and 63% (Experiment 3) of trials in their own experiments, has argued that fixation inaccuracies merely introduce noise to word recognition data. Unfortunately, for any experiments requiring fixation precision, it would be unsafe to imagine that fixation inaccuracies merely add noise because systematic biases in fixation to one side of the visual field are known to occur and even “random” fixation errors either side of a required fixation point can produce systematic left-right differences in the identifiably of words [63]. Accordingly, the principle adhered to in the present study was that the contribution of research to understanding influences of precise fixation location on word recognition can be known only if the precise location at which fixations occur can be identified.

2. Additional analyses of accuracy data in Experiment 1 were conducted using a linear mixed-effect model [103,104] specifying participants and stimuli as random effects, and lexicality, hemisphere, handedness, and experiment session as fixed factors. For foveal displays there was an effect of lexicality (b = .14, SE = .03, *t* = 4.63) but no effect of hemisphere (b = .03, SE = .03, *t* = 1.08), and no significant interactions involving hemisphere (*t* < 1). For extrafoveal displays there were main effects of lexicality (b = .08, SE = .03, *t* = 2.66) and hemisphere (b = .10, SE = .03, *t* = 3.06), and no significant interactions involving hemisphere (*t* < 1). These analyses therefore confirm that an LH-advantage is observed for stimuli displayed at extrafoveal but not foveal locations.

3. Additional analyses of error rates and reaction times in Experiment 2 were conducted using a linear mixed-effect model specifying participants and stimuli as a random effects, and lexicality, hemisphere, handedness, and experiment session as fixed factors. Error rates for foveal stimuli produced a main effect of lexicality (b = .15, SE = .03, *t* = 5.87) but no significant main effect of hemisphere (b = .01, SE = .03, *t* = .30) or interactions involving hemisphere (*t* < 1). Reaction times for correct responses to foveal stimuli produced a main effect of lexicality (b = 51.13, SE = 9.24, *t* = 5.53) but no significant main effect of hemisphere (b = 7.16, SE = 8.76, *t* = .82) or interactions involving hemisphere (*t* < 1). Error rates for extrafoveal stimuli produced a main effect of lexicality (b = .27, SE = .27, *t* = 9.93), a main effect of hemisphere (b = .09, SE = .27, *t* = 3.19), and an interaction between lexicality and hemisphere (b = .18, SE = .04, *t* = 4.74) that reflected an LH advantage for words but not pseudowords. Reaction times for correct responses to extrafoveal stimuli produced a main effect of lexicality (b = 59.41, SE = 9.81, *t* = 6.05), a main effect of hemisphere (b = 35.88, SE = 9.25, *t* = 3.88), and an interaction between lexicality and hemisphere (b = 31.45, SE = 13.74, *t* = 2.29), that reflected an LH-advantage for words but not pseudowords. Analyses using logistic regression produced the same pattern of results for error rates for foveal and extra-foveal presentations. Thus, these additional analyses confirmed an LH-advantage for words in extrafoveal but not foveal locations.

4. Computational approaches to split-foveal word recognition acknowledge the importance of exterior letters for word recognition and suggest that this is an emergent property of split-foveal word processing that reflects the history of fixating a word at various locations [22,105]. However, even these approaches recognise that split-foveal processing does not accommodate the privileged role that exterior letters have as a unified feature in word recognition and propose that low spatial-frequency information about the extremities of words must be projected bilaterally via sub-cortical routes.

References

96. Jordan TR, Paterson KB, Kurtev S, Xu M (2009) Do fixation cues ensure fixation accuracy in split-fovea studies of word recognition? Neuropsychologia 47: 2004-2007.

97. Paterson KB, Jordan TR, Kurtev S (2009) Binocular fixation disparity in single word displays. J Exp Psychol Human 35: 1961-1968.

98. Batt V, Underwood G, Bryden MP (1995) Inspecting asymmetric presentations of words differing in informational and morphemic structure. Brain Lang 49: 202–223.

99. Findlay JM, Kapoula Z (1992) Scrutinization, spatial attention, and the spatial programming of saccadic eye movements. Q J Exp Psychol-A: 633-647.

100. Jones B, Santi A (1978) Lateral asymmetries in visual perception with and without eye movements. Cortex 14: 164-168.

101. Sugishita M, Hamilton CR, Sakuma I, Hemmi I (1994) Hemispheric representations of the central retina of commissurotomized subjects. Neuropsychologia 32: 399-415.

102. Terrace HS (1959) The effects of retinal locus and attention on the perception of words. J Exp Psychol 58: 382-385.

103. Baayen, RH (2008) Analysing linguistic data: A practical introduction to statistics using R. Cambridge, UK: Cambridge University Press.

104. McCulloch CE, Searle, SR (2000) Generalized, Linear, and Mixed Models. New York: John Wiley and Sons.

105. Shillcock RC, Monaghan P (2001) The computational exploration of visual word recognition in a split model. Neural Comput 13: 1171-1198.
